# Student trainee and paid internship programs have positive results but do little to influence long-term employee diversity in the USDA forest service

**DOI:** 10.1371/journal.pone.0277423

**Published:** 2022-11-28

**Authors:** Michael J. Dockry, Sonya S. Sachdeva, Cherie L. Fisher, Laura S. Kenefic, Dexter H. Locke, Lynne M. Westphal

**Affiliations:** 1 Department of Forest Resources, University of Minnesota, St. Paul, MN, United States of America; 2 USDA Forest Service, Northern Research Station, Evanston, IL, United States of America; 3 USDA Forest Service, Northern Research Station, Orono, ME, United States of America; 4 USDA Forest Service, Northern Research Station, Baltimore, MD, United States of America; West Virginia University, UNITED STATES

## Abstract

Women and Black, Indigenous, and People of Color (BIPOC) employees are underrepresented in science and natural resource management institutions. Student and recent graduate trainee and internship programs have been used to try to address this in United States federal agencies over the last few decades. Our study evaluates how effective such programs are at improving U.S. federal workforce diversity. We used a comprehensive employee dataset from the United States Department of Agriculture (USDA) Forest Service–which has the largest natural resource management workforce in the country–to analyze the demographic characteristics and career paths of paid interns from 1996–2017. We found that a majority of employees who started as interns later converted to permanent employment with the USDA Forest Service. In addition, Black and Hispanic interns were, respectively, 5 and 3 times more likely than White interns to work for the agency in permanent positions after their internships. However, people who started as interns had significantly shorter USDA Forest Service careers than those who started in permanent positions. White women entering directly into permanent positions typically advanced to higher pay grades through promotion faster than White women who entered as interns. Finally, male BIPOC interns involuntarily separated (*i*.*e*., were fired) at significantly higher rates than all other employees. Our study suggests that while internship employment programs can be an effective tool for hiring a diverse workforce, they are not sufficient to close the overall workforce diversity gap. In addition, only a small percentage of new hires every year are interns. To achieve a level of representation that mirrors the civilian labor force, our study suggests that internship programs need to focus on long-term employee retention and be of significantly larger scale.

## Introduction

Underrepresentation of Black, Indigenous, and People of Color (BIPOC) employees in science, technology, engineering, and math (STEM) is a global phenomenon [1], and the United States is no exception [2–4]. This lack of racial, ethnic, and gender diversity is also seen within natural resource fields [5–15]. While federal agencies in the United States are required to have a workforce “that represents all segments of society” [16], underrepresentation persists in federal science agencies [6, 17, 18]. A diverse workforce has been shown to improve organizational performance [19–22] and innovation [23–25], and can result in better science [19, 26], but the goal of creating a diverse science workforce is often not achieved [27–30].

The need for diversity in science and government is multi-faceted. Riccucci and Van Ryzin [31] note that representation in a government’s workforce can increase trust in government services and policies, thereby increasing compliance and accountability. But lack of diversity can also affect the science conducted, the questions identified as important, and the approaches taken to address these questions [25, 32]. These same issues can hold true in ecological research. Examples include ongoing institutional and structural racism that hinders scientific and social progress [1, 33, 34], and “helicopter research” where researchers from Western countries drop into less-developed or post-colonial countries to take samples and collect information, often claiming “discovery” of species long known to locals, while providing few benefits to the country and people where the research was conducted [35, 36]. The same critiques can be found regarding research in the U.S. around environmental justice issues and more:

Right or wrong, research can drive decisions. If we do not address the power dynamic in the creation of research, at best, we are driving decision-making from partial truths. At worst, we are generating inaccurate information that ultimately does more harm than good in our communities. This is why we must care about how research is created. ([37], p.6)

Internships have been a component of U.S. federal workforce diversity strategies for decades [5, 10, 38–40]. These programs have been used to bring “high-quality individuals with limited work experience” into the federal government [41]. Internship programs that have been successful in recruiting and retaining diverse employees have usually contained a strong mentorship component [42], paid interns for their work [7, 43], and provided opportunities for growth and learning, meeting senior leaders, and working on group projects [41]. Internships have been particularly noted as important for diversifying natural resource fields in particular [43–45]. Several studies have looked at the importance of mentorship and internships within higher education. One study, for example, suggested that natural resource agencies can increase diversity by introducing minority students to natural resources careers [46]. Studies have also shown that men and underrepresented minority undergraduates who participate in mentored research projects had closer relationships with faculty mentors and higher scholarly productivity than women, White, and Asian undergraduates [47], students who participate in faculty mentored research enrolled in and completed post-graduate degrees at higher rates than students who don’t [48], and undergraduate research and mentorship programs in agricultural sciences have increased underrepresented students in agricultural degree programs [49, 50]. Additionally, women and underrepresented minority women who participate in research and internship programs have greater perception of their ability to perform engineering and technical tasks than those that don’t participate in internships [51]. While these studies showed undergraduate university programs benefit from mentorship and internships, there is surprisingly little literature on the effectiveness of these programs at diversifying STEM (Science, Technology, Engineering & Mathematics) fields beyond academia.

In this paper, we seek to understand the effectiveness of using paid internship programs—student and recent graduate trainee programs, in U.S. federal government parlance—to diversify the workforce of a natural resource agency, the USDA Forest Service. The USDA Forest Service is a decentralized, science-based land management agency [52–54], and it shares administrative procedures and hiring challenges with other land management agencies [18].

USDA Forest Service workforce diversity has been studied several times [e.g. 54–57]. Recently Westphal and others [17] presented longitudinal data on racial/ethnic and gender diversity for all USDA Forest Service employees by pay grade and branch of the agency between 1995 and 2017. Their findings were mixed with respect to changes in representation. For example, the overall number of agency employees decreased by nearly 20% during that period, with little increase in diversity outside of higher leadership levels (where the percentages of women and BIPOC employees increased over that period). In contrast, percentages of women in lower grades and in the National Forest System (the largest of the agency’s four branches) declined, as did the number of employees identifying as American Indian/Alaskan Native. Westphal and others [17] concluded that there are persistent problems with USDA Forest Service workforce diversification efforts that merit further investigation.

Using the same dataset, Sachdeva and others [58] assessed the relationship between race and gender and various metrics of career success in the USDA Forest Service. They found differences among demographic groups that suggest inequities in the agency’s hiring, promotion, and retention practices. Specifically, female employees of color advanced more slowly and had shorter careers than other groups despite starting at the agency at higher average pay grades. Male employees of color, on average, started work at the agency at lower pay grades than people in other groups. Both male and female employees of color were more likely to be terminated (*i*.*e*., fired), as opposed to resigning voluntarily or retiring. In contrast, White male employees were promoted most rapidly and had the longest careers.

The lack of progress in achieving a diverse workforce in the USDA Forest Service is not due to a lack of awareness of the issue. Beginning in the 1970s and continuing through the present, there have been concerted efforts to increase employee diversity in the agency [57, 59–63]. Student trainee and recent graduate internship positions have been one tool used to try to increase diversity. It can often be easier for federal agencies to hire interns and then convert them to permanent positions than to create and fill permanent positions with open searches. Federal government interns are paid, which avoids the pitfall that unpaid internships are inherently discriminatory since not everyone can afford to work without pay just to gain experience [7]. Internships within the federal government are often perceived to be pathways to long-term careers. Federal internships are thought to be used during times when budget constraints or federal government policies make it difficult or impossible to hire permanent employees through regular channels [39]. Prior to 2010, there were numerous student and intern programs for federal agencies, including the USDA Forest Service, such as the Student Temporary Employment Program (STEP), Student Career Employee Program (SCEP), Presidential Management Fellowship (PMF), and others [64]. In 2010, Executive Order (E.O.) 13562 consolidated federal student and recent graduate trainee and internship programs into the Pathways Programs [65]. The Pathways Programs recognized that the federal government benefits from a “diverse workforce that includes students and recent graduates” and that the government is required to “achieve a workforce that represents all segments of society as provided in 5 U.S.C. 2301(b)(1)” [65].

This paper aims to provide an empirical assessment of the effectiveness of internships as an approach to achieving workforce diversity. This is an important gap to fill, given the prevalence of internships as a tool to achieve diversity and the lack of empirical assessments of their effectiveness. This research analyzed USDA Forest Service employee data to compare the career trajectories of those who started in internship programs with those who did not. Specific research questions were:

- What are the race/ethnicity and gender (hereafter, demographic) profiles of USDA Forest Service student and recent graduate trainees (interns)?
- Are employees who begin their careers with the USDA Forest Service as interns more likely to stay in permanent positions than those who enter the USDA Forest Service via other hiring routes?
- Finally, how does beginning as an intern relate to other career trajectory metrics, such as length of service, career advancement, initial pay grade level, and separation type (manner of leaving the agency)?

Addressing these questions helps us understand the effectiveness of internships as a diversification tool and may suggest ways to improve such programs. The USDA Forest Service and other government agencies need evidence-based assessments to guide their diversification efforts in order to achieve a representative, highly effective workforce.

## Methods

### Dataset

The research and analyses described in the current manuscript do not utilize primary data from human participants. Rather, we used a fully anonymized dataset provided to us by the USDA Forest Service Human Resource Management department. These employment data were anonymized before the research team had access to them. The data included variables such as race/ethnicity (self-reported by employees), gender (self-reported by employees as binary male/female), Deputy Area (branch of the agency either National Forest System, Research and Development, State and Private Forestry, Business Operations, and the Office of the Chief), job series (type of occupational group either. communications, administrative, forest resources, and facilities), appointment type (e.g. temporary, permanent, intern/trainee), and separation (how an employee departed the agency when applicable) for every fiscal year (FY) from 1995 to 2017. These files were reshaped into a dataset containing one record per employee per FY [66].

We identified interns by querying the job series field. Intern positions include all Pathways positions after 2013 and others like STEP, SCEP, and Cooperative Education positions before 2013 [67]. Presidential Management Fellows (the U.S. federal internship program for graduate students and recent graduates with advanced degrees) were also included as interns throughout the entire timeframe. Due to the limitations of the dataset, this paper only tracks employees who were classified as “99” (intern/trainee) and does not track employees who were students but not classified in internship positions (e.g., a non-intern/trainee seasonal summer recreation technician). Employees who were hired into non-intern permanent positions are called “career conditional” in U.S. federal government parlance and referred to as “permanent” throughout the rest of the paper.

To understand the demographics of interns who became permanent USDA Forest Service employees, we used a subset of the dataset that included only employees who had permanent positions at some time in their USDA Forest Service careers [58]. To assess career trajectories for USDA Forest Service employees who came into the agency as interns compared to those who were not interns, we used a subset of the dataset of permanent employees for whom we could see their entire career trajectory (*i*.*e*., employees for whom we have information on how they started and left the agency). This is necessary to analyze the full arc of an employee’s career. This data subset includes employees who began their career in 1996 (the year our dataset begins) or later and left the agency (voluntarily resigning, retirement, or termination, collectively called separation) before 2017 (the year the dataset ends).

### Analysis

We first used summary statistics to report percentages and changes over time in the demographic make-up of the USDA Forest Service workforce. We also used a binomial generalized linear modeling approach, implemented in R [68], to see if there were significant differences in conversion from intern to permanent employment based on race/ethnicity, gender, deputy area, and whether the intern was brought on through the Pathways Programs or one of the earlier internship programs. The R function to carry out this analysis was: glm(formula = EverPerms ~ Enter99 * BIPOC * Gender + deputy_area + job_field + Min_FY3, data = FirstYearAllEmps, family = binomial (link = "logit")). We combined African American, American Indian, Asian, Hispanic, Native Hawaiian/Pacific Islander, and “two or more” races into one category (titled BIPOC) to create more even group sizes for statistical analysis. This approach has been accepted in other recent literature [1] despite clear drawbacks stemming from painting heterogenous groups with a broad stroke. Minoritized groups are so rare in these data that collapsing into coarse categories was necessary for any statistical analyses. For our analysis of interns’ and permanent employees’ career trajectories, we used a subset of the dataset that includes only employees whose tenure was encompassed by the years 1996 and 2017. For this analysis we also limited the permanent employees to those who were hired at General Service Levels (GS) 1 through 7. We made this decision because these are the typical grade levels for newly hired employees and 98% of interns were first hired at GS 1–7 levels (see Office of Personnel Management (OPM) https://www.opm.gov/policy-data-oversight/pay-leave/pay-systems/general-schedule/ for more information about GS scales). The mean comparisons for these analyses were conducted using ANOVAs implemented in R [66]. The following R functions were used to calculate Initial Grade, Length of Service, and Advancement, respectively: Initial grade, aov(data = Grades.df.Seps, Initial.Grade ~ BIPOC*Gender*Enter99 + deputy_area + job_field + Min_FY3); Length of Service, aov(data = Grades.df.Seps, FiscalYearsActive ~ BIPOC*Gender*Enter99 + deputy_area + job_field + Min_FY3); and Advancement, aov(data = Grades.df.Seps, Advance ~ BIPOC*Gender*Enter99 + deputy_area + job_field + Min_FY3).

## Results

### Profiles of USDA forest service interns

**Fig 1:**
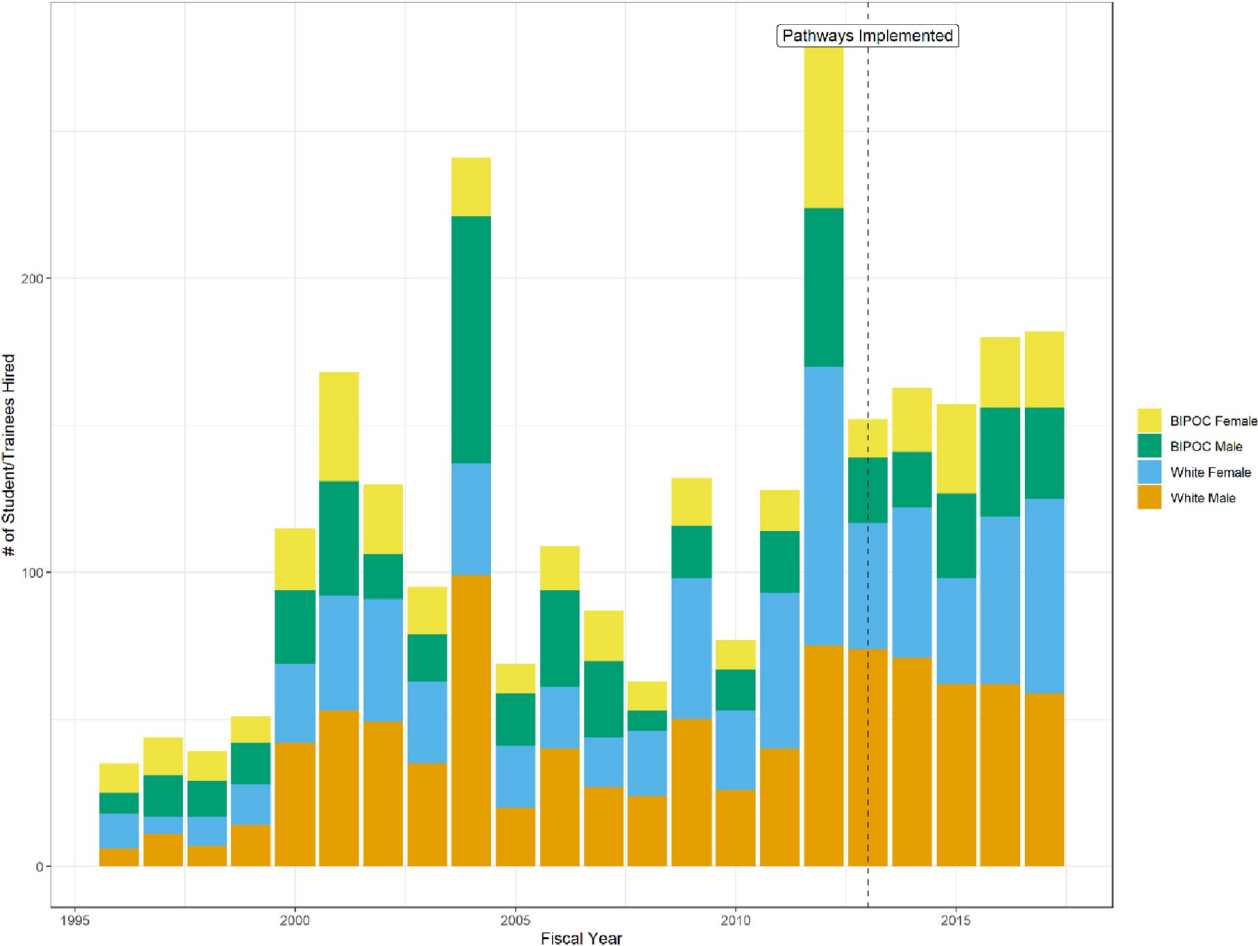
Number of student and recent graduate trainees entering USDA forest service between 1996 and 2017.

Using the selection criteria described above, 2,697 employees were hired as interns (2% of all hires) between 1996 and 2017. The number of interns entering the USDA Forest Service increased and became more consistent (averaging over 150 per year) after the Pathways Programs started in 2013 (Fig 1). Overall, USDA Forest Service hiring of both permanent employees and interns decreased after 2013; however, hiring decreased by a factor of 4 for permanent employees compared to a significantly lower factor of 2 for interns (Table 1). The vast majority (98%) of USDA Forest Service employees began their careers as permanent employees and not as interns (Table 2). Of those who entered as interns, the greatest number were White men, followed by White women, Hispanic men, Hispanic women, African American men and women, Asian men, Asian women, and finally American Indian men and American Indian women. Overall, less than 3% of employees who report their race as White, American Indian, or Two or More started with the USDA Forest Service as interns compared to 10% of African American, 8% of Native Hawaiian/Pacific Islander, 7% of Asian, and 6% of Hispanic employees (Table 2). In other words, African American and Hispanic employees were, respectively, 5 and 3 times more likely than White employees to join the USDA Forest Service as interns. In fact, BIPOC employees accounted for more than a third of the total employees who entered the agency as interns (Table 3). By comparison, White employees made up an overwhelming majority of employees entering the USDA Forest Service in non-intern permanent positions, while BIPOC employees made up less than 15% (Table 3).

**Table 1:** Analysis of hiring trends for number of permanent and trainee employees before and after 2013 implementation of the pathways programs.

	Pre-2013	Post-2013
Permanent employee	94242	20683
Trainee	1863	834

X-squared = 293.72, df = 1, p-value < 2.2e-16

**Table 2:** Race/ethnicity and gender composition(number and percent) of all USDA forest service employees by permanent and trainee from 1996–2017.

Gender	Race/Ethnicity	Permanent Employee		Trainee	
Female		n	%	n	%
	African American	1256	3%	153	13%
	American Indian/Alaskan Native	840	2%	36	3%
	Asian	633	2%	48	4%
	Hispanic	1778	4%	148	12%
	Native Hawaiian/Pacific Islander	30	0%	3	0%
	Two or More	573	1%	35	3%
	White	34466	87%	773	65%
Male					
	African American	1597	2%	153	10%
	American Indian/Alaskan Native	1465	2%	47	3%
	Asian	876	1%	64	4%
	Hispanic	4266	6%	248	17%
	Native Hawaiian/Pacific Islander	67	0%	6	0%
	Two or More	1573	2%	37	2%
	White	65493	87%	946	63%

*Note*. Columns sum to 100% within Genders

**Table 3:** Composition of employees, by race and gender, who enter in a permanent versus trainee position (1996–2017).

Race/Ethnicity Gender	Position Type	
	Permanent employees	Trainees
BIPOC Female	4%	16%
White Female	30%	29%
BIPOC Male	9%	20%
White Male	57%	35%

*Note*. Columns sum to 100% within Position Type

### Intern transitions to permanent positions

As noted above, one goal of U.S. federal internship positions and the Pathways Programs is to convert interns to permanent employment within the agency. Of the 2,697 interns who entered the USDA Forest Service between 1996 and 2017, 1,701 (63%) became permanent employees. Looking across all employees, we fit a binomial generalized linear regression model predicting conversion to a permanent position using whether employees entered through an internship program, gender, race/ethnicity (combined into BIPOC), deputy area, job field, and the year of initial employment (to control for temporal trends) (Table 4). These factors predicted about 13% of the variance in total conversion rates (Adjusted R^2^ = .13, F(13, 17608) = 1020, *p* < .001).

**Table 4:** Generalized linear model estimates predicting likelihood of conversion to a permanent position across all employees in the USDA forest service by trainee program, race/ethnicity, gender, deputy area, job field, and year of first employment with the agency. Deputy Area refers to branches within the agency, akin to different departments. Job Field pertains to an individual’s type of occupation.

Effect	Estimate	*SE*	t value	*p*
Intercept	6.08	0.41	14.77	< .001
Student/Trainee Program	0.27	0.02	12.32	< .001
BIPOC (White)^a^	-0.07	0.01	-10.93	< .001
Gender (Male)^b^	0.04	0.01	5.21	0.00
Deputy Area (Bisops & Finance)^c^	0.35	0.01	56.99	< .001
Deputy Area (Office of the Chief)^c^	0.36	0.01	26.27	< .001
Deputy Area (Research & Development)^c^	-0.03	0.00	-6.36	0.00
Deputy Area (State & Private Forestry)^c^	0.33	0.02	17.61	< .001
Job Field (Admin)^d^	0.26	0.00	60.61	< .001
Job Field (Communications)^d^	0.25	0.01	32.73	< .001
Job Field (Facilities)^d^	0.12	0.00	24.37	< .001
Year Hired	0.00	0.00	-14.19	< .001
Student/Trainee X BIPOC (White)	0.03	0.03	0.95	0.34
Student/Trainee X Gender (Male)	0.04	0.03	1.48	0.14
BIPOC (White) X Gender (Male)	0.02	0.01	2.82	0.00
Student/Trainee X BIPOC (White) X Gender (Male)	-0.05	0.04	-1.43	0.15
Observations 117,364 Adjusted R^2^ = 0.13				

*Note*: ^a^ White relative to BIPOC.

^b^Male relative to Female.

^c^ All deputy areas relative to the National Forest System.

^d^ All job fields relative to Forest Resources.

^e.^ Student/Trainee Programs are relative to all other ways of entering the Forest Service (e.g., as a volunteer, or through a partner agency).

**Fig 2:**
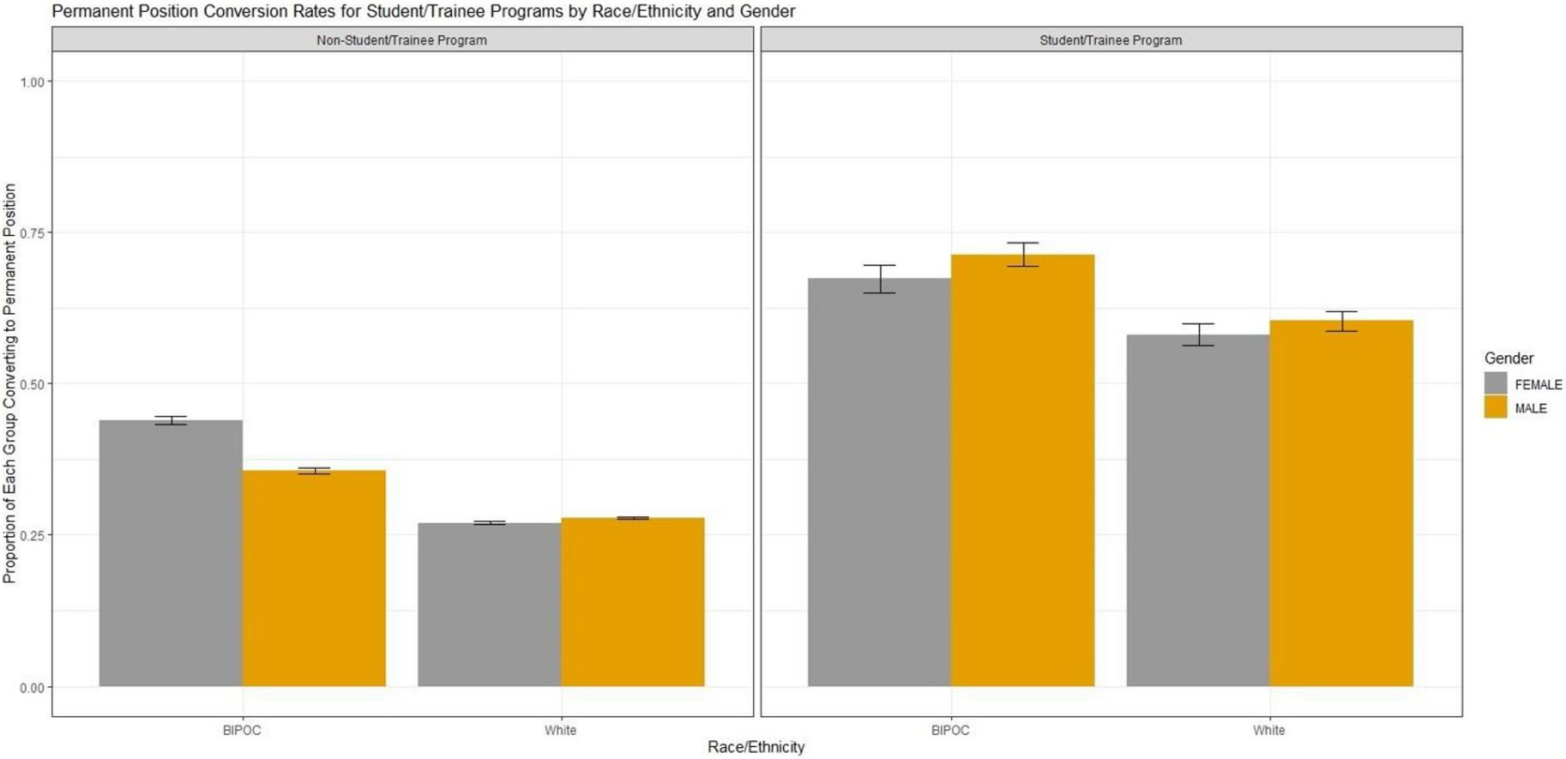
Conversion to permanent employee positions for employees entering through the student and recent graduate trainee program versus other routes, race/ethnicity and gender.

Our data show that converting to a permanent position regardless of entry route became less common over the years encompassed by the dataset (β_InitialYear_ = -.003, *p* < .001). However, even while controlling for these trends, we find that people who entered the USDA Forest Service through an internship program were more likely to convert to a permanent position than people who entered the agency through a different route, β_Intern_ = .27, *p* < .001 (see Fig 2). Importantly, our data do not show an interaction between entering as an intern and race/ethnicity or gender, indicating that the internship program does not confer a higher likelihood of converting to a permanent position based on either of those demographic variables. However, as shown in previous work [17], an inherent advantage of being male (β_male_ = .04, *p* < .001) and particularly, White and male β = .02, *p* < .05), exists in attaining a permanent position with the USDA Forest Service. White men have a 13% greater likelihood than other groups of converting to a permanent position.

Further analyses delved deeper into the characteristics of the interns themselves. Looking within this group, the model showed a significant relationship between conversion to permanent positions as a function of race/ethnicity (combined into BIPOC), gender, deputy area, job field, and whether the intern was brought on through the 2013 Pathways Programs and/or earlier internship program (Table 5). Altogether, these variables explained 42% of the variance in the rates of conversion to permanent positions (adjusted R^2^ = .42, F(11, 2685) = 180.3, *p* < .001). The results for this analysis suggest that BIPOC interns were more likely to convert to permanent employment than White interns (β_White_ = -.06, *p* = .01). Additionally, interns working in the National Forest System deputy area (the largest branch of the agency) were less likely to convert to permanent positions than interns working in the smaller branches including Business Operations & Finance, the Office of the Chief, and State &Private Forestry. However, Research & Development was an exception (β_R&D_ = -.41, *p* < .001). Interns hired in the *pre*-Pathways era were also more likely to convert to permanent employment than interns who started after the Pathways Programs began (β_Pathways_ = .61, *p* < .001). Gender and the interaction between gender and race/ethnicity did not have significant effects.

**Table 5:** Generalized linear model estimates predicting likelihood of conversion from an trainee to a permanent position in the Forest Service by race/ethnicity, gender, deputy area, job field, and pre/post Pathways programs.

Effect	Estimate	*SE*	t value	*p*
Intercept	0.269	0.023	11.841	< .001
BIPOC (White)^a^	-0.057	0.022	-2.550	0.011
Gender (Male)^b^	-0.001	0.024	-0.057	0.954
Deputy Area (Bisops & Finance)^c^	0.102	0.030	3.354	0.001
Deputy Area (Office of the Chief)^c^	0.253	0.042	6.071	< .001
Deputy Area (Research & Development)^c^	-0.405	0.030	-13.287	< .001
Deputy Area (State & Private Forestry)^c^	0.190	0.055	3.443	0.001
Job Field (Admin)^d^	-0.135	0.020	-6.850	< .001
Job Field (Communications)^d^	0.094	0.042	2.267	0.023
Job Field (Facilities)^d^	0.038	0.021	1.802	0.072
Pathways^e^	0.609	0.016	38.694	< .001
BIPOC X Gender	0.015	0.030	0.508	0.611
Observations 2,697 Adjusted R^2^ = 0.422				

*Note*: ^a^ White relative to BIPOC.

^b^Male relative to Female.

^c^ All deputy areas relative to the National Forest System.

^d^ All job fields relative to Forest Resources.

^e^ Pre-Pathways relative to post-pathways.

### Intern career trajectories

Employees who started their careers as interns had different career trajectories as seen in metrics for initial grade, career advancement, length of service (time with the agency), and separation. Of the interns who transitioned to career positions, our dataset contains 1,198 employees for whom we have data on their entire career trajectory/tenure with the USDA Forest Service (meaning we can see if they left the agency through voluntary or involuntary separation). Compared to permanent employees starting with the agency at grades between GS-1and GS-7, a typical range of entry-level permanent positions, employees who started as interns were hired into slightly lower grade levels (Table 6). Female interns, both BIPOC and White, advanced in grade level at rates similar to permanent employees but BIPOC male interns advanced at a slower rate than employees entering with permanent appointments. Interns who converted to permanent employment also had a significantly (*p* < .05) shorter mean length of service than employees who started in permanent positions across gender and race/ethnicity categories: 2.97 years less for BIPOC women, 4.34 years less for White women, 4.67 years less for BIPOC men, and 4.44 years less for White men (Table 6). Additionally, BIPOC male employees (both those entering as interns and those entering in permanent positions) were more likely to be involuntarily separated (*i*.*e*. fired) than those in any other employee category, in some cases more than twice as likely. Finally, White male interns who converted to permanent employment were statistically more likely to be involuntarily separated than White men who started in permanent positions.

**Table 6:** Beginning career type influences on initial grade, LOS, advancement, and separation.

Career Type	Race/Ethnicity Gender	Initial* Grade	Length of Service	Grades/Year Advancement	Rate of Involuntary Separation
Permanent	BIPOC Female	4.77^a^	7.34 ^a^	0.23^a^	5.9%^a^
	White Female	4.51 ^b^	8.71^b^	0.27^b^	4.0%^a^
	BIPOC Male	4.10 ^c^	8.99^b^	0.25 ^a^	10.2%^b^
	White Male	4.13 ^c^	9.71^c^	0.30^c^	4.8%^a^
Trainee	BIPOC Female	4.15 ^c^	4.37^d^	0.23 ^a^	6.0%^a^
	White Female	4.26 ^c^	4.80 ^d^	0.29^a^	5.0%^a^
	BIPOC Male	3.78^d^	4.32 ^d^	0.18^d^	10.6%^b^
	White Male	4.06 ^c^	5.27 ^d^	0.27^a^	7.2%^c^

Note: 1) Permanent Employees’ initial grades were limited to those starting at GS1-7, typical grades for initial employment. 2) All cells with different letters in a column are statistically distinct from each other at *p* < .05 based on pairwise t-tests.

## Discussion

We assessed the demographic profiles of USDA Forest Service student and recent graduate trainees (*i*.*e*. paid interns), whether those profiles changed with the consolidation of internship programs in 2013, and the conversion of interns to permanent positions. We also looked at the career trajectories of employees starting as interns versus employees starting in permanent positions. This work is an extension of our analysis of diversity in the USDA Forest Service, where we found declining diversity overall, and significant demographic disparities in career trajectories. For example, BIPOC women have been slower to advance even though they are often hired in at higher grade levels than others, and BIPOC men are more likely to be terminated than other subsets of the workforce [58].

### Intern profiles

Student and recent graduate internship positions are one tool the USDA Forest Service has used to increase diversity within the agency. While overall hiring of permanent employees decreased after 2013, the year the internship programs were consolidated into Pathways Programs, intern hiring also decreased but at a rate two times lower than the decrease in permanent hires. While our data do not answer why this happened, it may be the result of the agency using the Pathways Programs to maintain workforce numbers at a time when hiring permanent employees was severely curtailed [39]. The overall decrease in US federal government hiring has been linked to widening racial workforce gaps [69]. Hiring a diverse workforce is an explicit goal of the Pathways Programs [67] and in this regard, the program appears successful.

When combining our data to look at BIPOC employees by gender, greater percentages (but not overall total numbers) of BIPOC men and BIPOC women enter as interns compared to the percentages entering directly as permanent employees. The opposite is true, however, for White employees. Thus, internship positions are extremely important for hiring BIPOC employees into the agency. However, the total number of White interns converting to permanent positions is 27% greater than all BIPOC interns converting to permanent employees combined (Table 2). Additionally, in contrast to anecdotal reports that agency managers lost hiring flexibility with the consolidation of the Pathways Programs in 2013, there has been a significantly greater number of interns entering the USDA Forest Service since that time. Despite the importance of internship positions as entry points for BIPOC employees, BIPOC intern hires are so few in number (n = 978 over our entire dataset) compared to the number of employees in the agency (n = 117,622 total from 1996–2017) that the current numbers and trends in hiring interns will never be sufficient to close the agency’s diversity gap (see 17,18).

### Intern transitions to permanent employment

Internships are important for long-term diversity when interns are converted to permanent employees. In this regard, higher percentages of male and female BIPOC interns were converted to permanent employees than male and female White interns. However, due to lower total numbers of BIPOC interns than White interns and despite the higher percentages of conversions to permanent employment, BIPOC internship numbers are not sufficient to reduce the diversity gap in the agency if current hiring patterns continue. In other words, even though male BIPOC interns are 11% more likely to convert to permanent positions than male White interns, there are still 44% fewer male BIPOC interns overall. Additionally, there appears to be an important difference in conversion to permanent employment with the transition to the Pathways Program starting in 2013. Interns hired in the pre-Pathways era were more likely to convert to permanent positions than interns hired under the Pathways Programs from 2013–2017. This result could be a function of time as Pathways was implemented only four years before our data end in 2017 and there were fewer permanent positions overall for interns to move into, but it is worth tracking this trend to understand the implications regarding long-term agency diversity. Finally, interns working in the National Forest System were less likely to convert to permanent positions than interns in other areas of the agency. One explanation for this finding could be the greater use of internship appointments (both pre/post Pathways) on the national forests for what amount to essentially temporary summer jobs.

### Intern career path and separation

Interns entered permanent employment at slightly lower grade levels than employees who entered directly as permanent employees. Because interns started in the agency at lower grade levels, there was more room for them to advance than permanent employees who started at slightly higher grades. The reason for this is that they were being trained to move up in the agency and many of the interns who were students received a college degree during their employment period qualifying them for higher grade levels. This holds true for male BIPOC interns who advanced significantly more than all the other demographic groups, both as trainees and as permanent employees. White male interns also followed this trend and advanced more than White male permanent employees. This is not the case, however, for all groups, and in fact White women in permanent positions advanced more than White women who entered as interns. Consistent with Sachdeva and others [58], these trends clearly show that male employees advanced differently in their USDA Forest Service careers than female employees.

Retention is also an important component of diversity and inclusion efforts. In this regard, employees who started as interns had significantly shorter lengths of service (about half as long) across their entire tenure than employees who started in permanent positions. This was true regardless of gender or race/ethnicity. Our study is not able to identify the reasons for the shorter length of service but there could be several explanations. Interns who have a portion of their education paid for by the agency are required to work a certain amount of time at the agency or pay back the cost of the agency support. Once this obligation is fulfilled and the intern has worked the required amount of time, they may decide to leave the agency. One study on university students who worked in internship positions showed that only 60% of interns converted to permanent positions and if the intern did not plan on working long-term with the organization, there was little that could be done to recruit them into permanent positions [70]. Also, younger employees have been shown to leave U.S. federal employment at higher rates than older employees due to several factors including pay, meaningfulness of the work, and work-life balance [71].

Another explanation could be that the agency climate is such that interns who converted to permanent positions, especially women and BIPOC employees [54, 62], soon left the agency for other opportunities. This does not fully explain why White male interns also had shorter lengths of service. Workplace climate can be inferred in our study by looking at involuntary separations. In this regard, male BIPOC interns and permanent employees were involuntarily separated at significantly higher rates than all other employee categories. This has been shown in studies looking at the U.S. Postal Service, another U.S. federal agency, as well [72, 73]. Additionally, in our study, White men who started as interns were involuntarily separated at greater rates than all other groups except BIPOC men.

These trends are difficult to explain with our data; however, it could be that male employees are being separated for inappropriate behavior that the USDA Forest Service has been trying to control for decades [see 62, 74, 75]. It is unclear why BIPOC men are involuntarily separated at higher rates than all others, but studies have shown inequities between BIPOC and White employees in promotion practices and rates of termination related to an unwelcome climate and discrimination [see 76, 77]. Black men have been shown to be fired at higher rates than other racial groups in the U.S. Postal Service [72, 73]. This trend could be a reflection of systemic racism [78]. Regardless of the reason for these trends, the shorter length of service and higher involuntary separations for BIPOC men reduce the impact that the student trainee and recent graduate internship programs can have on the agency’s diversity.

## Conclusion

There is scant research on student internships and their impact on workforce diversity in STEM and natural resource organizations. This study explores the impacts of a student and recent graduate internship program and the implications for diversity within a U.S. federal government agency. The USDA Forest Service has been working to diversify the agency’s workforce for decades and internship programs have been one of the key strategies used to move towards this goal. We examined employment data from 1996–2017 and show that internship programs have been successful at bringing diverse employees into the agency, but retention of these employees has been less successful. This suggests that the USDA Forest Service may not be creating and sustaining a workplace climate that supports long-term retention of interns. Thus, internship programs have not been sufficient to diversify the agency to meet the stated goal to “achieve a workforce that represents all segments of society” as provided in 5 U.S.C. 2301(b)(1)” (61). At all career stages for employees entering as interns (hiring, conversion to permanent employment, career trajectory, and separation) there are not enough interns to close the diversity gap since only about 2.2% of all new hires are interns. While our study illuminates demographic and career trends for interns and newly hired permanent employees, there is a need for more research to understand the reasons for shorter lengths of service and the higher rates of involuntary separation for men. Our study has implications for both federal agencies and other organizations. It suggests that the scale of internship programs needs to be large if they are going to be successful at diversifying an organization’s employees. It also suggests that additional work is needed to create a climate where interns can develop into successful long-term employees.
